# Analysis of emergency calls achieved in a French emergency dispatching centre: what resources for which patients

**DOI:** 10.1186/cc12212

**Published:** 2013-03-19

**Authors:** O Tilak, J Cuny, N Assez, P Goldstein, E Wiel

**Affiliations:** 1 Lille University Hospital Center, Lille Cedex, France

## Introduction

Following patients referred by the French emergency dispatching centre (SAMU 15) in different hospital services (emergency or ICU) is not always easy. The purpose of this study was to analyze each call arriving in the emergency dispatching centre, define resources for transportation, the host hospital department, and analyze the short-term outcome of these patients.

## Methods

We conducted a prospective, observational study, by collecting all data recorded at the emergency dispatching centre in a continuous 24-hour period. Each call creating a medical file was included. We identified the type of call, various administrative information, the purpose of call, resources triggered, medical advice, hospital department admission, and clinical evolution.

## Results

A total of 877 patients were included; 67.80% calling from home, 15.63% calling from a public place. Firemen ambulances were sent in 38.89%, private ambulance in 24.65% and an emergency medical ambulance (SMUR) in 11.23%. Simple medical advice without transportation was given in 13.19% of cases. In total, 22.18% of patients were entrusted to the family; 2.89% of patients refused transportation; 69.94% of triggered firemen ambulances were done by a centre 18 call, without any medical regulation by an emergency physician. A total of 68.59% patients were referred to the emergency department, 1.2% in ICU, and 1.8% in cardiac ICU. In the emergency room, 50.78% of patients received a simple medical consultation with biological analyses, and then returned home. In total, 25.50% of patients were hospitalized in a medical or surgical department, and 12.42% in the short-term hospitalised unit of the emergency department (stay duration <24 hours). Some 5.10% of patients worsened and were oriented in the ICU. A total 3.77% of patients in a cardiac ICU. In total, 73.84% of patients had stay duration less than 6 hours in the ED, 24.45% <24 hours. Forty percent of patients supported by firemen and 54% supported by private ambulance left the hospital after a single medical consultation.

## Conclusion

Nearly 70% of patients calling the French emergency medical dispatching centre are sent to hospital. Those transportations are supported for two-thirds of cases by a private ambulance or firemen ambulance. One out of two patients only receive a simple medical consultation in the ED, and go back home. This may concur to the deficiency of using general medicine in town. They prefer using emergency services for free. Only one patient out of four was hospitalized more than 24 hours.

